# A Smartphone-based Application for Self-Management in Multiple Sclerosis

**DOI:** 10.1155/2021/6749951

**Published:** 2021-06-16

**Authors:** Mahdieh Mokhberdezfuli, Haleh Ayatollahi, Abdorreza Naser Moghadasi

**Affiliations:** ^1^Department of Health Information Management, School of Health Management and Information Sciences, Iran University of Medical Sciences, Tehran, Iran; ^2^Multiple Sclerosis Research Center, Neuroscience Institute, Tehran University of Medical Sciences, Tehran, Iran; ^3^Health Management and Economics Research Center, Iran University of Medical Sciences, Tehran, Iran

## Abstract

**Background:**

Multiple sclerosis (MS) is a chronic inflammation of the central nervous system and self-management is necessary for MS patients. The purpose of the present study was to develop a smartphone-based application for self-management in multiple sclerosis.

**Methods:**

This research was conducted in two phases. In the first phase, users' requirements were investigated by using a questionnaire. The participants were 120 MS patients and six neurologists. In the second phase, a prototype of the application was designed and its usability was evaluated by using QUIS questionnaire.

**Results:**

Most of the proposed educational content, data elements, and the application functions, such as medication time reminder, assessing the severity of fatigue, and calculating the score of the Fatigue Severity Scale were found necessary to be included in the application. Finally, the usability of the application was evaluated by the users and the average of mean values was 7.6 out of 9 which indicated a “good” level of user satisfaction.

**Conclusions:**

The application designed in this study was able to collect patient data and facilitated consulting physicians at the point of need. It is expected that the patients' quality of life and health status can be improved by using this application. However, more research is required to investigate the efficiency and effectiveness of this application in terms of reducing the number of visits to the medical centers, improving self-management skills of MS patients and their quality of life.

## 1. Introduction

Multiple sclerosis (MS) is an autoimmune disease which can affect the central nervous system (CNS) and may cause a variety of symptoms. It is also the most common debilitating neurological disorder in adults. Not only MS is regarded as a serious health problem but also its economic burden in terms of the costs associated with the patient care and treatments as well as patients' reduced economic activities have received more attention in the past years [1–3].

It is worth mentioning that the treatment of chronic neurological diseases such as MS may follow a different protocol from other chronic periodic diseases. MS patients have persistent disorders that require continuous monitoring and management, and healthcare services are often provided by multiple healthcare professionals in diverse situations. In addition, MS patients require understanding their disorders in order to be able to better manage them. As there is currently no cure for MS, the life of these patients is sometimes affected by multiple disabilities for the rest of their lives. In addition, the unpredictable and complex nature of the disease makes managing conditions more difficult and tedious. Therefore, self-management is of great importance in the life of MS patients [4, 5].

Self-management is a dynamic, interactive, and routine process to manage chronic illnesses. It refers to an individual's ability to manage symptoms, treatments, lifestyle changes, and health status as well as social, psychological, and cultural difficulties [6]. The self-management program is a set of routine activities to assist patients with chronic diseases. It helps patients to get actively involved in controlling factors affecting their health status, monitoring symptoms, and making appropriate decisions to manage the complexities of their disease. The use of self-management programs in various chronic diseases has been successful and not only has improved patient health status but also has helped physicians in managing pains, reducing depressive symptoms, and taking timely actions [7].

Self-management requires special skills and trainings in different situations [8]. It has been recognized as a sustainable and low-cost intervention and can have a major impact on the public health. In particular, for patients with chronic diseases, self-management has significant benefits such as improving their health status and quality of life [9].

Self-management of a chronic disease, such as MS, can be defined as a dynamic and an active coping process [10]. The results of the study conducted by Ghahari et al. showed that self-management in MS patients had positive impacts in terms of the patients' psychological and lifestyle improvement [5]. Therefore, self-management can be regarded as a potential approach that helps to control the symptoms associated with MS disease. However, this intervention can be provided in different ways and requires further investigations to be improved [11–13].

Recently, the use of telehealth services has made a significant progress in managing many chronic diseases, such as diabetes, cancer, and hypertension. For example, as smartphones are used on a daily basis and are easy to use, individuals' clinical conditions can also be tracked and reported on this device instantly [14]. Other benefits of using a smartphone in healthcare services include proper distribution of resources, reduction of economic burden, and promotion of patient care [15]. Mobile-based applications can also be used for training, rehabilitating, and monitoring patients' symptoms and treatment [16]. The results of different studies revealed that more than 85% of MS patients have mobile phones, and most of them use it to manage their disease [17, 18]. Although many applications, such as Basic MS Explorer [19], MS Care Connect [20], and MS 101.me [21], are now available for MS patients, some of them have not been successful in meeting patients' and healthcare professionals' requirements. In fact, each application has its own strengths and weaknesses [14, 22]. Some applications have either limited educational content or healthcare professionals have not approved them, and some others have focused on measuring a limited number of symptoms, such as fatigue, anxiety, or cognitive impairments [5]. Therefore, it seems that an application is needed that can bring together a number of functions that are not presently available in a single application. In the current study, we aimed to develop a smartphone-based application for self-management in multiple sclerosis which can support the information needs of patients and provide them with their required functions.

## 2. Materials and Methods

This research was conducted in 2020 and composed of two phases. In the first phase, 6 neurologists who worked in three teaching hospitals and 120 MS patients completed a questionnaire to determine the educational content, data elements, and functions required in the application. The questionnaire was designed based on the literature review [3, 12, 23–29] and consisted of four main sections and 74 items. The first section of the questionnaire addressed the personal characteristics of the participants (3 items), and the second section consisted of the educational content (34 items), data elements (23 items), and functions (14 items) required for the application. The educational content included general information about MS (6 items), MS patient's lifestyle (5 items), symptoms management (17 items), MS treatment (3 items), and characteristics of the physical environment and place of residence (2 items). The data elements required for the application included individual (10 items) and clinical data (13 items). The participants were asked to determine which item is “necessary” or “unnecessary.” At the end of each part, an open-ended question was considered for the participants to add any new items from their point of views. The face and content validity of the questionnaire was assessed by five neurologists. The reliability of the questionnaire was also assessed by calculating the internal consistency measure (KR-20 = 0.94).

To analyze the data obtained from the first phase of the research, descriptive statistics was used and frequency distributions, mean values, and standard deviations were calculated using SPSS software, version 26. In order to include the most important educational content and items in the application, other similar studies [30] were reviewed and a cutoff point of 60% was considered. It means that if 60% of the participants or more agreed on the necessity of an item in the first phase of the study, that item would be included in the application; otherwise, it would be removed.

In the second phase, a prototype of the smartphone-based application was designed based on the results derived from the first phase of the study. Initially, a number of books and related literature were used to extract educational contents [3–7, 9–14]. Then, other parts of the application were designed, and finally, QUIS questionnaire [31] was used to evaluate the usability of the application [32]. The participants of this phase were patients (*n* = 60) and neurologists (*n* = 6). It is notable that the neurologists and patients who took part in the second phase of the study were different from those who participated in the first phase.

The application was installed on the patients' smartphones and they worked with it. Then, they were requested to complete QUIS questionnaire. This questionnaire consisted of six sections as follows: personal information (3 items), overall reaction to the software (6 items), screen (4 items), terminology and system information (6 items), learning (6 items), and system capabilities (5 items). This questionnaire was designed based on a 10-point Likert scale, and its validity and reliability have already been reported in other studies (*α* = 0.94) [31]. To analyze the data related to the second phase of the research, the mean value of each item of the questionnaire was calculated and classified in three levels of “poor” (0–3), “average” (3.1–6), and “good” (6.1–9), and descriptive statistics was used to analyze data.

## 3. Results

As noted before, 6 neurologists and 120 patients participated in the first phase of the study. In terms of age, the highest frequency belonged to the age range of 41–50 years old (*n* = 5, 83.3%) for neurologists (42.5 ± 3.3) and 31–40 years old (*n* = 57, 47.5%) for patients (35.7 ± 8.2). The majority of patients (*n* = 93, 77.5%) and half of the neurologists (*n* = 3, 50%) were female. In terms of education, the highest frequency (*n* = 49, 40.9%) was related to the patients who had a high school diploma or less education. According to the neurologists' and patients' perspectives, most of the items mentioned in the questionnaire were found necessary. As a result, they were included in the application.  Table 1 presents the educational content, data elements, and functions of the application.

Based on the results derived from the first phase of the study, a prototype of the application was designed. It was written using Basic4Android programming language, and SQLite was used to create the database. The application could be used by patients and physicians.

### 3.1. Patient's Panel

**Figure 1:**
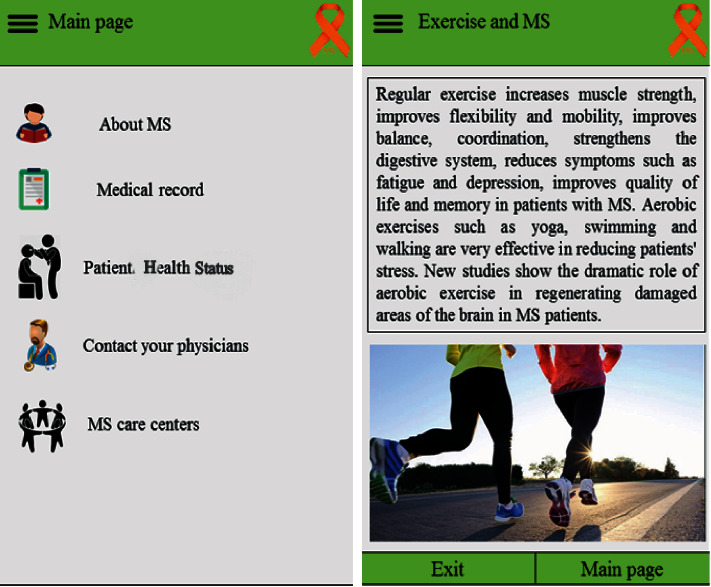
Patient's panel.

The patient's panel included five main sections, namely, educational content related to MS (about MS), patient medical record, patient health status, contacting a physician, and MS care centers. After logging into the application, a patient could enter his/her personal and clinical data to create a medical record. Patient health status consisted of five sections: medications, medication time reminder, patient's symptoms, patient's stress and anxiety, and patient's fatigue. A patient could also send messages to his/her physician and receive a response. Moreover, basic information related to MS care centers including their addresses and telephone numbers was available in this panel (Figure 1).

### 3.2. Physician's Panel

**Figure 2:**
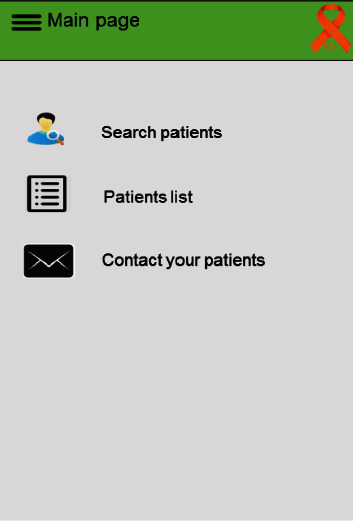
Physician's panel.

The physician's panel included three main sections: search patients, patients list, and contact your patients (Figure 2). By choosing “search patient,” the physician could find a patient by entering his/her national ID number, name, or surname. Then, the physician could be able to view the patient's medical record, medications, current symptoms, and the level of fatigue and stress along with the messages sent by the patient. In the patients list, the physician could check the list of patients that entered their information into the application. The physician could also communicate with the patient through the application by selecting the patient's name, writing the message, and sending it in response to the patient's query.

After designing the application, 60 MS patients and six neurologists participated in the second phase of the study to evaluate the usability of the application. Among patients, the highest frequency was related to the age range of 31–40 years old (36.8 ± 10.3, *n* = 21, 35%), and half of the neurologists were within the same age range (41.6 ± 4.6, *n* = 3, 50%). Furthermore, the majority of patients (*n* = 49, 81.7%) and half of the neurologists (*n* = 3, 50%) were female. In terms of education, the highest frequency (*n* = 21, 35%) was related to the patients with a bachelor's degree. The results of the usability evaluation are presented in Table 2.

As Table 2 shows, the mean values for different sections of the questionnaire were between 6.1 and 9, indicating that the application was evaluated at a “good” level by the users.

## 4. Discussion

Self-management in chronic diseases is directly related to increasing the quality of life, reducing disabilities, and decreasing treatment costs. MS patients usually require a wide range of self-management skills, as they may experience constant changes in their health status [12]. Currently, technology is changing the process of health management, and the use of computer-based applications is increasing drastically. So far, several smartphone- , tablet- , and mobile-based applications have been developed to support people in managing their health status, chronic diseases, or certain aspects of health [23, 24]. Similarly, advances in mobile technology have been used to create mHealth applications that support patients in self-management of their chronic diseases [25, 26].

In the current study, a smartphone-based application was developed for self-management in multiple sclerosis. In this application, educational contents including the general information about MS and patient's lifestyle, symptoms management, MS treatment, physical environment, and place of residence were provided. Moreover, necessary individual and clinical data elements and application functions were considered based on the results of the first phase of the study. Similarly, in a study conducted by Kafami et al., the demographic data and a health status questionnaire, which included the subsections of general health, daily activities, physical problems, mental problems, pain, fatigue, emotional status, emotional functioning, social functioning, and perception of health were used [27]. In the present study, patients were able to enter their latest health status into the application and their daily conditions were saved in the application.

In Madani et al.‘s study, the required individual data and MS complications were collected using a questionnaire. A self-care program was set and patients were educated about MS symptoms, treatment methods, complications, and symptom management skills in the bladder and bowel disorders, constipation, sensory disorders, memory problems, fatigue, muscle cramps, and exercise. A booklet was also given to the patients in each session [28]. However, in the present study, the required educational contents, which were similar to the contents proposed in Madani et al.‘s study, were included in the smartphone-based application.

In a study conducted by D'Hooghe et al., the main objective was to assess the level of fatigue among MS patients after training and providing rehabilitation programs. The researchers developed a smartphone-based application which measured fatigue as one of the most reported and common complaints of MS patients [29]. Similarly, in the present study, two of the most widely used questionnaires, namely, the fatigue severity scale [33] and the hospital anxiety and depression scale [34] were included in the application. The score of the questionnaires was sent to the physician and she/he could assess the patient's fatigue and anxiety status.

The results of Salimzadeh et al.‘s study revealed that most of the existing MS self-management applications have concentrated on the self-management of symptoms, diagnosis and treatment, medication management, communication between patients, increasing patients' awareness, providing news of conferences and meetings, and donations to MS communities [35]. It should be noted that most applications deal with only one specific aspect of MS self-management. For instance, some applications purely follow training purposes [36], while others focus on assessing patient's fatigue and anxiety level [37, 38]. In the present study, an attempt was made to develop an application which included most of the features of other similar applications in a single application to be able to meet users' requirements.

Finally, the usability of the application was evaluated and patients were requested to express their opinions about their overall reaction to the application, screen design, terminology and educational content, learning capabilities, and overall functions of the application. The findings revealed that the application usability and user satisfaction were at a good level. Similarly, in a study conducted by Tacchino et al., patients completed a nine-item questionnaire to evaluate the usability of the application after eight weeks of using it [39]. However, in the present study, due to the limitations caused by the prevalence of Covid-19 disease, MS patients were asked to evaluate the usability of the application by completing the questionnaire in the clinic environment, where they were waiting to visit their physicians.

## 5. Limitations of the Study

Although the application designed in the current study could be useful in the self-management of MS patients, more evaluation studies are required to improve it. It is notable that the second phase of the study was performed during Covid-19 pandemic. Therefore, the number of MS patients that referred to the clinics was very limited and many patients were reluctant to participate in the study to respect the health and safety protocols. As a result, conducting future evaluation studies with more patients is recommended. Moreover, in the first phase of the study, the highest frequency of patients had a high school diploma or less education. However, in the second phase of the study, the highest frequency was related to the patients with a bachelor's degree. As the content of the application was provided based on the results of the first phase of the study, it was expected that different group of users with a variety of education levels could use the application. Although the application was evaluated at a good level, conducting more evaluation studies with different groups of patients in terms of age and education is recommended.

## 6. Conclusions

The aim of this study was to develop a smartphone-based application for MS self-management. Considering that these types of applications require to be designed based on the real needs of end-users and the opinions of healthcare providers, users' requirements were investigated before designing the application. The designed application was also evaluated by the users and the results showed that they were satisfied with that. As MS is a chronic disease and patients have a significant role in disease management, using such an application can help reducing patients' visits to the hospitals and medical centers, saving time, and decreasing heathcare costs. Improving the health status of patients by continuous monitoring and remote supervision of physicians will be other benefits of using this application. However, further studies on the impact of this application on the quality of life of MS patients as well as the cost-effectiveness of this application are recommended.

## Figures and Tables

**Table 1:** Educational content, data elements, and functions of the application.

Educational content	General information about MS (MS definition, different types of MS, MS risk factors, MS symptoms, MS complications, and MS and pregnancy), MS patient's lifestyle (nutrition, exercise, and stress management), symptoms management (muscle weaknesses, movement problems, vision problems, sensory symptoms, speech disorders, urinary dysfunction, cognitive disorders, fatigue, depression, sexual disorders, facial muscle spasms, temperature sensitivity, headache and dizziness, electric shock feeling in the body, attack symptoms, myokymia, trigeminal neuralgia), MS treatment (different types of MS treatments, MS medications, and side effects), and physical environment and place of residence (suitable place of residence and suitable weather for the patient)

Individual data elements	Name, surname, age, marital status, national ID number, sex, and contact number

Clinical data elements	History of MS among first degree relatives, onset of the first MS symptoms, date of MS diagnosis, number of hospital admissions for attacks, walking problems (asking patients if they had any problem with walking), MS medications used over the past five years, year of medication initiation, year of medication discontinuation, drug allergy, and cause of drug discontinuation

Application functions	Describing patient general condition, (describing current symptoms and details ofan attack), medication time reminder, reminders for medications that are close to run out, describing current medications and their doses, consulting a physician by sending messages, completing the Hospital Anxiety and Depression Scale bythepatient andcalculating its score, assessing the severity of fatigue and calculating the score of the Fatigue Severity Scale, sending messages to the patient by her/his physician, searching patient information by physician, and introducing MS care centers.

**Table 2:** The results of the usability evaluation.

Usability evaluation	Mean + SD
Overall reaction to the software	7.6 ± 1.0
Screen	8.1 ± 0.8
Terminology and system information	7.9 ± 1.0
Learning	7.2 ± 1.0
System capabilities	7.5 ± 1.1

## Data Availability

All data generated or analyzed during this study are included in this published article.

## Supplementary Materials

Supplementary Materials Both questionnaires used in the current study are uploaded as supplementary files. .
