# Supplementary material for: A Smartphone-based Application for Self-Management in Multiple Sclerosis
Source: J Healthc Eng. 2021 Jun 16;2021:6749951. doi: 10.1155/2021/6749951 (PMC8225446; doi:10.1155/2021/6749951)
Supplement: Supplementary Materials — Both questionnaires used in the current study are uploaded as supplementary files. . [file 6749951.f1.docx]

**Questionnaire 1- Necessary content for the application**

**Section 1:** **Personal information**

1. Age:
2. Sex: Male 🞎 Female 🞎
3. Education: High school diploma or less educated 🞎 Associate degree 🞎

Bachelor’s degree 🞎 Master’s degree 🞎 Ph.D 🞎

**Section 2:** **Necessary content for the application**

Please insert a check mark (🗸) for the items that you think they are necessary to be included in the application.

| **NO** | **Educational content** | | **Necessary** | **Unnecessary** |
| --- | --- | --- | --- | --- |
| 4 | General information about MS | MS Definition |  |  |
| 5 |  | Different types of MS |  |  |
| 6 |  | MS risk factors |  |  |
| 7 |  | MS symptoms |  |  |
| 8 |  | MS complications |  |  |
| 9 |  | MS and pregnancy |  |  |
| 10 | MS Patient’s lifestyle | Nutrition |  |  |
| 11 |  | Exercise |  |  |
| 12 |  | Stress management |  |  |
| 13 |  | Smoking |  |  |
| 14 |  | Hookah |  |  |
| 15 | Symptoms management | Muscle weaknesses |  |  |
| 16 |  | Movement problems |  |  |
| 17 |  | Visual problems |  |  |
| 18 |  | Sensory symptoms |  |  |
| 19 |  | Speech disorders |  |  |
| 20 |  | Urinary dysfunction |  |  |
| 21 |  | Cognitive disorders |  |  |
| 22 |  | Depression |  |  |
| 23 |  | Fatigue |  |  |
| 24 |  | Sexual disorders |  |  |
| 25 |  | Facial muscle spasms |  |  |
| 26 |  | Temperature sensitivity |  |  |
| 27 |  | Headache and dizziness |  |  |
| 28 |  | electric shock feeling in the body |  |  |
| 29 |  | Attack symptoms |  |  |
| 30 |  | Myokymia |  |  |
| 31 |  | Trigeminal neuralgia |  |  |

| **NO** | **Educational content** | | **Necessary** | **Unnecessary** |
| --- | --- | --- | --- | --- |
| 32 | MS treatment | Different types of MS treatments |  |  |
| 33 |  | MS Medications |  |  |
| 34 |  | MS Medications side effects |  |  |
| 35 | Physical environment and place of residence | Suitable place of residence |  |  |
| 36 |  | Suitable weather |  |  |
| 37 | If there are any more items please add them here. | | | |
| **Data elements** | | | | |
| 38 | Individual data | Name |  |  |
| 39 |  | Surname |  |  |
| 40 |  | Age |  |  |
| 41 |  | Father’s name |  |  |
| 42 |  | Marital status |  |  |
| 43 |  | National ID number |  |  |
| 44 |  | Sex |  |  |
| 45 |  | Place of birth |  |  |
| 46 |  | Address |  |  |
| 47 |  | Contact number |  |  |
| 48 | Clinical data | History of MS among first degree relatives |  |  |
| 49 |  | History of MS among second degree relatives |  |  |
| 50 |  | History of MS among other relatives |  |  |
| 51 |  | Onset of the first MS symptoms |  |  |
| 52 |  | Date of MS diagnosis |  |  |
| 53 |  | Number of hospital admissions for attacks |  |  |
| 54 |  | Walking problems |  |  |
| 55 |  | MS medications used over the past five years |  |  |
| 56 |  | Year of medication initiation |  |  |
| 57 |  | Year of medication discontinuation |  |  |
| 58 |  | Drug allergy |  |  |
| 59 |  | Cause of medication discontinuation |  |  |
| 60 | If there are any more items please add them here. | | | |

| **NO** | **Application functions** | **Necessary** | **Unnecessary** |
| --- | --- | --- | --- |
| 61 | Describing patient general condition |  |  |
| 62 | Describing current symptoms and details of an attack |  |  |
| 63 | Medication time reminder |  |  |
| 64 | Reminders for medications that are close to run out |  |  |
| 65 | Describing current medications |  |  |
| 66 | Describing medication doses |  |  |
| 67 | Consulting a physician by sending messages |  |  |
| 68 | Completing the Hospital Anxiety and Depression Scale by patient and calculating its score |  |  |
| 69 | Assessing the severity of fatigue and calculating the score of the Fatigue Severity Scale |  |  |
| 70 | Sending messages to the patient by her/his physician |  |  |
| 71 | Determining the urgency for a patient visit |  |  |
| 72 | Searching patient information by physician |  |  |
| 73 | Introducing MS care centres |  |  |
| 74 | If there are any more items please add them here. | | |

**Questionnaire for User Interface Satisfaction**

**Personal information**

1. Age:
2. Sex: Male 🞎 Female 🞎
3. Education: High school diploma or less educated 🞎 Associate degree 🞎

Bachelor’s degree 🞎 Master’s degree 🞎 Ph.D 🞎

| OVERALL REACTION TO THE SOFTWARE | |  | 0 | 1 | 2 | 3 | 4 | 5 | 6 | 7 | 8 | 9 |  |
| --- | --- | --- | --- | --- | --- | --- | --- | --- | --- | --- | --- | --- | --- |
| 1. | System performance | terrible |  |  |  |  |  |  |  |  |  |  | wonderful |
| 2. | Difficulty with using the system | difficult |  |  |  |  |  |  |  |  |  |  | easy |
| 3. | Your feeling about the system | frustrating |  |  |  |  |  |  |  |  |  |  | satisfying |
| 4. | System power | inadequate power |  |  |  |  |  |  |  |  |  |  | adequate power |
| 5. | Working with the system | dull |  |  |  |  |  |  |  |  |  |  | stimulating |
| 6. | System flexibility | rigid |  |  |  |  |  |  |  |  |  |  | flexible |
| SCREEN | |  | 0 | 1 | 2 | 3 | 4 | 5 | 6 | 7 | 8 | 9 |  |
| 7. | Reading characters on the screen | hard |  |  |  |  |  |  |  |  |  |  | easy |
| 8. | Highlighting simplifies task | not at all |  |  |  |  |  |  |  |  |  |  | very much |
| 9. | Organization of information | confusing |  |  |  |  |  |  |  |  |  |  | very clear |
| 10. | Sequence of screens | confusing |  |  |  |  |  |  |  |  |  |  | very clear |
| TERMINOLOGY AND SYSTEM INFORMATION | |  | 0 | 1 | 2 | 3 | 4 | 5 | 6 | 7 | 8 | 9 |  |
| 11. | Use of terms throughout system | inconsistent |  |  |  |  |  |  |  |  |  |  | consistent |
| 12. | Terminology related to task | never |  |  |  |  |  |  |  |  |  |  | always |
| 13. | Position of messages on screen | inconsistent |  |  |  |  |  |  |  |  |  |  | consistent |
| 14. | Prompts for input | confusing |  |  |  |  |  |  |  |  |  |  | clear |
| 15. | Computer informs about its progress | never |  |  |  |  |  |  |  |  |  |  | always |
| 16. | Error messages | unhelpful |  |  |  |  |  |  |  |  |  |  | helpful |
| LEARNING | |  | 0 | 1 | 2 | 3 | 4 | 5 | 6 | 7 | 8 | 9 |  |
| 17. | Learning to operate the system | difficult |  |  |  |  |  |  |  |  |  |  | easy |
| 18. | Exploring new features by trial and error | difficult |  |  |  |  |  |  |  |  |  |  | easy |
| 19. | Remembering names and use of commands | difficult |  |  |  |  |  |  |  |  |  |  | easy |
| 20. | Performing tasks is straightforward | never |  |  |  |  |  |  |  |  |  |  | always |
| 21. | Help messages on the screen | unhelpful |  |  |  |  |  |  |  |  |  |  | helpful |
| 22. | Supplemental reference materials | confusing |  |  |  |  |  |  |  |  |  |  | clear |
| SYSTEM CAPABILITIES | |  | 0 | 1 | 2 | 3 | 4 | 5 | 6 | 7 | 8 | 9 |  |
| 23. | System speed | too slow |  |  |  |  |  |  |  |  |  |  | fast enough |
| 24. | System reliability | unreliable |  |  |  |  |  |  |  |  |  |  | reliable |
| 25. | System tends to be | noisy |  |  |  |  |  |  |  |  |  |  | quiet |
| 26. | Correcting your mistakes | difficult |  |  |  |  |  |  |  |  |  |  | easy |
| 27. | Designed for all levels of users | never |  |  |  |  |  |  |  |  |  |  | always |
